# Network-based co-expression analysis for exploring the potential diagnostic biomarkers of metastatic melanoma

**DOI:** 10.1371/journal.pone.0190447

**Published:** 2018-01-29

**Authors:** Li-xin Wang, Yang Li, Guan-zhi Chen

**Affiliations:** 1 Department of Dermatology, The Affiliated Hospital of Qingdao University, Shandong, China; 2 Institute of Dermatology and Skin Hospital, Chinese Academy of Medical Sciences, Peking Union Medical College, Nanjing, China; University of Alabama at Birmingham, UNITED STATES

## Abstract

Metastatic melanoma is an aggressive skin cancer and is one of the global malignancies with high mortality and morbidity. It is essential to identify and verify diagnostic biomarkers of early metastatic melanoma. Previous studies have systematically assessed protein biomarkers and mRNA-based expression characteristics. However, molecular markers for the early diagnosis of metastatic melanoma have not been identified. To explore potential regulatory targets, we have analyzed the gene microarray expression profiles of malignant melanoma samples by co-expression analysis based on the network approach. The differentially expressed genes (DEGs) were screened by the EdgeR package of R software. A weighted gene co-expression network analysis (WGCNA) was used for the identification of DEGs in the special gene modules and hub genes. Subsequently, a protein-protein interaction network was constructed to extract hub genes associated with gene modules. Finally, twenty-four important hub genes (*RASGRP2*, *IKZF1*, *CXCR5*, *LTB*, *BLK*, *LINGO3*, *CCR6*, *P2RY10*, *RHOH*, *JUP*, *KRT14*, *PLA2G3*, *SPRR1A*, *KRT78*, *SFN*, *CLDN4*, *IL1RN*, *PKP3*, *CBLC*, *KRT16*, *TMEM79*, *KLK8*, *LYPD3* and *LYPD5*) were treated as valuable factors involved in the immune response and tumor cell development in tumorigenesis. In addition, a transcriptional regulatory network was constructed for these specific modules or hub genes, and a few core transcriptional regulators were found to be mostly associated with our hub genes, including GATA1, STAT1, SP1, and PSG1. In summary, our findings enhance our understanding of the biological process of malignant melanoma metastasis, enabling us to identify specific genes to use for diagnostic and prognostic markers and possibly for targeted therapy.

## Introduction

Melanoma, also known as malignant melanoma, is a type of cancer that develops from cells with different degrees of pigmentation, including melanotic and amelanotic melanocytes [1]. The main reason for the onset of melanoma is a low pigment content in skin exposed to ultraviolet light [2]. Metastatic melanoma is uncommon but aggressive, with high mortality rates [3], and is the most dangerous type of skin cancer. In the global context, there were 23.2 million new skin cancer cases in 2012 [2]. In 2015, there were 3.1 million people suffering from active disease, resulting in 59,800 deaths [4]. Globally, the incidence of melanoma is estimated to be between 3% and 7%; based on these estimates, the incidence of melanoma will double every 10–20 years [5].

When human skin is exposed to ultraviolet radiation (UVR), melanin formation is initiated by DNA strand breaks, thymidine dinucleotide fragments and the oxidation of the amino acid tyrosine [6]. Melanin is produced in special organelles called melanosomes contained in specific cells known as melanocytes [7]. The morphology and characteristics of melanosomes are important features that distinguish melanocyte-derived melanotic or amelanotic types of malignant melanomas [8]. Melanin can effectively dissipate 99.9% of the absorbed UVR [9]. Because of this property, melanin is thought to protect skin cells from UVR damage and reduce the risk of cancer. However, metastatic melanoma has been almost impossible to cure until recently, when, due to some important publications, metastatic melanoma has become the most promising evidence-based example of personalized medicine. Two of the most effective techniques for the treatment of metastatic melanoma have been identified, targeted therapy and checkpoint suppression [10,11]. These discoveries provide the basis for the use of next-generation sequencing techniques and immunobiology.

Recently, in addition to the simple extraction of information about differentially expressed genes, innovative biological methods (e.g., gene chips) have been applied to the screening of large amounts of genetic information. Additional analyses of the data identified a series of genes, including MAGE, GRP19, BCL2A1, SOX5 and BUB1, involved in the progression of metastatic melanoma [12]. In addition, transcriptome gene expression analysis is important for screening for abnormally expressed genes, and for revealing the genomic locations of the genes [13]. Furthermore, the method of weighted co-expression network analysis (WGCNA) has been applied to the information mining of malignant melanoma data. WGCNA, by constructing gene networks, can explain gene expression data. A gene network that is connected to highly co-expressed genes can be divided into different gene modules involved in individual functions [14]. In these gene modules, WGCNA can identify core-related genes, the so-called hub genes. These modules and their key genes may be involved in important pathological processes and may, therefore, have important clinical application as biomarkers or therapeutic targets for early diagnosis and evaluation of prognosis.

Although the success of recent single-agent and combination therapies is encouraging, there is still much work to be done to treat patients with appropriate stratification, to understand and circumvent treatment resistance and to identify new therapeutic targets. To improve our understanding of the biological pathology of metastatic melanoma, we analyzed the existing gene expression data of malignant melanomas using advanced network analysis strategies to explore the pathogenesis of metastatic melanoma.

## Materials and methods

### Analyses of gene expression data and differentially expressed genes

We used global mRNA expression profiles (level 3) from the National Institutes of Health (NIH), Genomic Data Commons database (GDC) with a total of 471 samples, including 103 primary solid tumors and 368 metastatic melanoma samples. The R 3.4 software and the Bioconductor packages were used to process the data collected from the GDC database. The background correction, quantile normalization and expression value calculations were performed using a robust multi-array averaging algorithm. The differentially expressed genes (DEGs) were extracted by the edgeR package, which implements an accurate statistical method of multigroup experiments developed by Robinson and Smyth [15] and provides statistical procedures for assessing the differential expression of RNA-Seq data in ChIP-Seq experiments. In this study, a log2 fold change (logFC) > 1 and P < 0.01 were selected as the screening standard for DEGs.

### Weighted gene co-expression network analysis

Co-expression networks were constructed with the identified DEGs. We first calculated the Pearson correlations between all genes present in the dataset and obtained the correlation matrix of the whole gene correlation. The power beta was then used to remove the weakly correlated genes while retaining the stronger ones. The process produced a weighted network called 'adjacent matrices' that was converted to a topological overlapping matrices (TOM) network. After constructing the TOM network, we used hierarchical, clustering genes to make the cluster dendrogram with branches corresponding to the gene co-expression modules. The module definition in the dendrogram was executed using the dynamicTreeCut algorithm with the following parameters: deep split = 2, maximum cut height = 0.95, minimal module size = 90 genes [16].

### Functional analysis of network module genes

To determine the biological mechanisms of melanoma alteration, the up-regulation and down-regulation of DEGs were used for Gene Ontology (GO) biology process terminology and Kyoto Encyclopedia Gene and Genomes (KEGG) pathway enrichment analysis. All the DEGs annotated in the GO database were used to classify GO functions through the clusterProfiler package and to understand the distribution of gene functionality at the global level. DEGs for KEGG enrichment analysis were mapped to the KEGG database. After the analysis of GO and KEGG data, significant selection criteria were set as false discovery rate (FDR) and P-value under a level of 0.05.

### PPI network construction

The online database resource search tool (STRING) for retrieving interactive genes provides protein-protein interaction information, including the prediction and comparison of inter-genomic interactions [17]. Protein-protein interaction (PPI) research can reveal the protein functions of DEGs at the molecular level and can explain the cellular mechanisms by elucidating the interactions of genome-wide proteins [18]. In this study, a PPI network of all identified DEGs was established. The up-regulation and down-regulation of the gene-encoded proteins are represented by nodes of different shapes and colors in the network, and lines represent the interactions between proteins. The widely linked genes (hub genes) in the PPI network are thought to be more closely related to most proteins.

### Transcriptional regulation network

In this study, to understand the transcriptional regulation of the hub genes, we used the University of California Santa Cruz (UCSC) database (http://genome.ucsc.edu/) TF-to-target to search for the interactions between a transcription factor (TF) and the hub genes and to assess the effect of a TF on the expression of the central gene. We downloaded the tfbsConsSites and tfbsConsFactors files from UCSC. The tfbsConsSites gives all the TF coordinate information, and the tfbsConsFactors site gives the TF identification information. We can predict the potential target relationships of all TFs based on the refGene file from UCSC and obtain the gene location information for hg18. There are 199 TF and 199,950 TF to the target interactions.

## Results

### Pre-processing of datasets

To improve the reliability of the analysis, microarray data containing 471 3-level expression data were pretreated and combined into two global datasets of both primary tumor tissue (TP) and melanoma malignant metastatic tumor (MT) tissue obtained from the GDC database. There were no statistically significant differences in the general backgrounds of the 103 cases of TP and 368 MT, including age, gender, race, ethnicity and vital status (**Table 1**).

**Table 1:** Characteristics of malignant melanoma patients.

Characteristics		MT (n = 368)	TP (n = 103)	P_value
Age, Mean(SD)		56.29(15.70)	64.71(13.96)	0.057
Gender	Female	138	42	0.259
	Male	230	61	
Race	NE	8	2	0.054
	Asian	5	7	
	Black or African American	1	0	
	White	354	94	
Ethnicity	NE	8	5	0.126
	Hispanic Or Latino	7	4	
	Not Hispanic Or Latino	353	94	
Vital Status	NA	1	0	0.345
	Alive	221	93	
	Dead	146	10	
Clark Level	NA	121	28	0.017
	I	6	0	
	II	17	1	
	III	63	14	
	IV	129	39	
	V	32	21	
AJCC Pathologic Tumor Stage	NA	34	4	0.958
	I/II NOS	13	1	
	Stage 0	7	0	
	Stage I	29	1	
	Stage IA	18	0	
	Stage IB	28	1	
	Stage II	26	4	
	Stage IIA	14	4	
	Stage IIB	19	9	
	Stage IIC	15	49	
	Stage III	39	2	
	Stage IIIA	15	1	
	Stage IIIB	35	12	
	Stage IIIC	55	12	
	Stage IV	21	3	
Submitted Tumor Site	NA	50	2	0.067
	Extremities	156	41	
	Head and Neck	30	8	
	Other Specify	9	4	
	Trunk	123	48	

NE: Not Evaluated, NA: Not Available, MT: malignant tumor, TP: primary tumor

AJCC: American Joint Committee on Cancer

### Detection of differentially expressed genes

**Fig 1:**
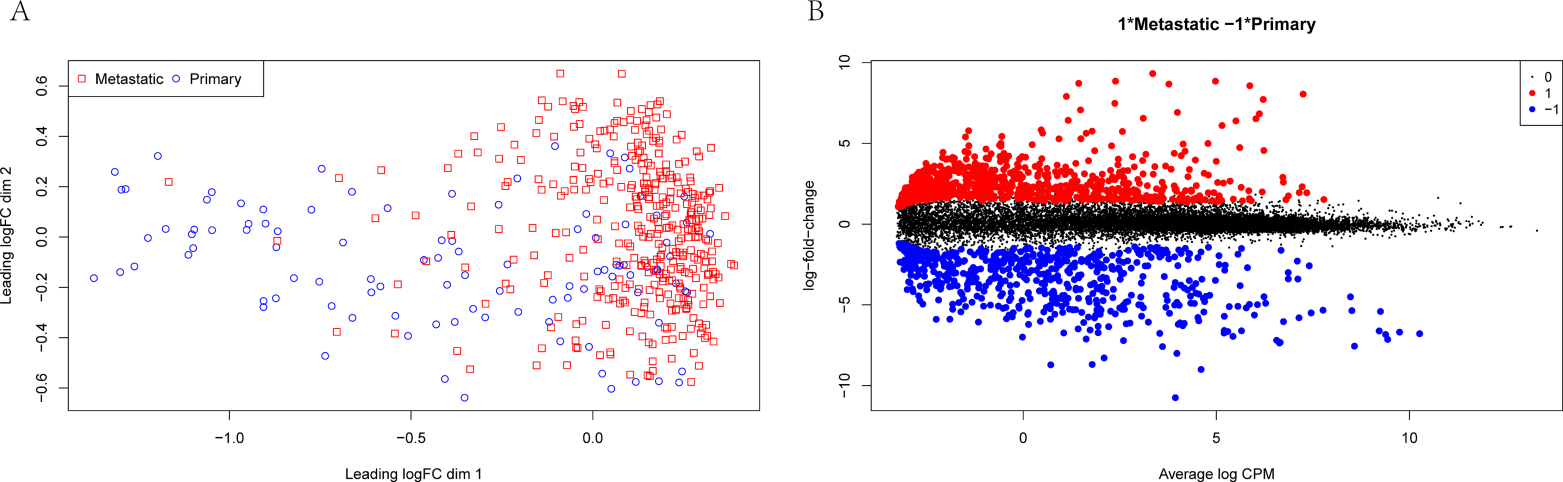
Differentially expressed genes (DEGs) for RNA-Seq data. **A.** Principal component analysis of the 471 samples. Each red symbol represents a metastatic melanoma sample, while the blue represents the primary tumor sample. **B:** Volcano plot of differentially expressed miRNAs. In the volcano plots, each color dot represents a down-regulated or up-regulated mRNA; red indicates genes with high levels of expression, and blue indicates genes with low levels of expression, above and below the median, respectively).

Principal component analysis (PCA) was considered to be able to explain the variance of the data and reveal the internal structure of the data. In the present study, the distribution of 471 samples was divided into two clusters (**Fig 1A**). A total of 1,568 DEGs was detected in this study. Compared with primary solid tumor samples, 639 down-regulated genes and 929 up-regulated genes were identified in the metastatic melanoma samples (P-value < 0.01, log2 fold change > 1) (**Fig 1B**).

### A weighted gene co-expression network

**Fig 2:**
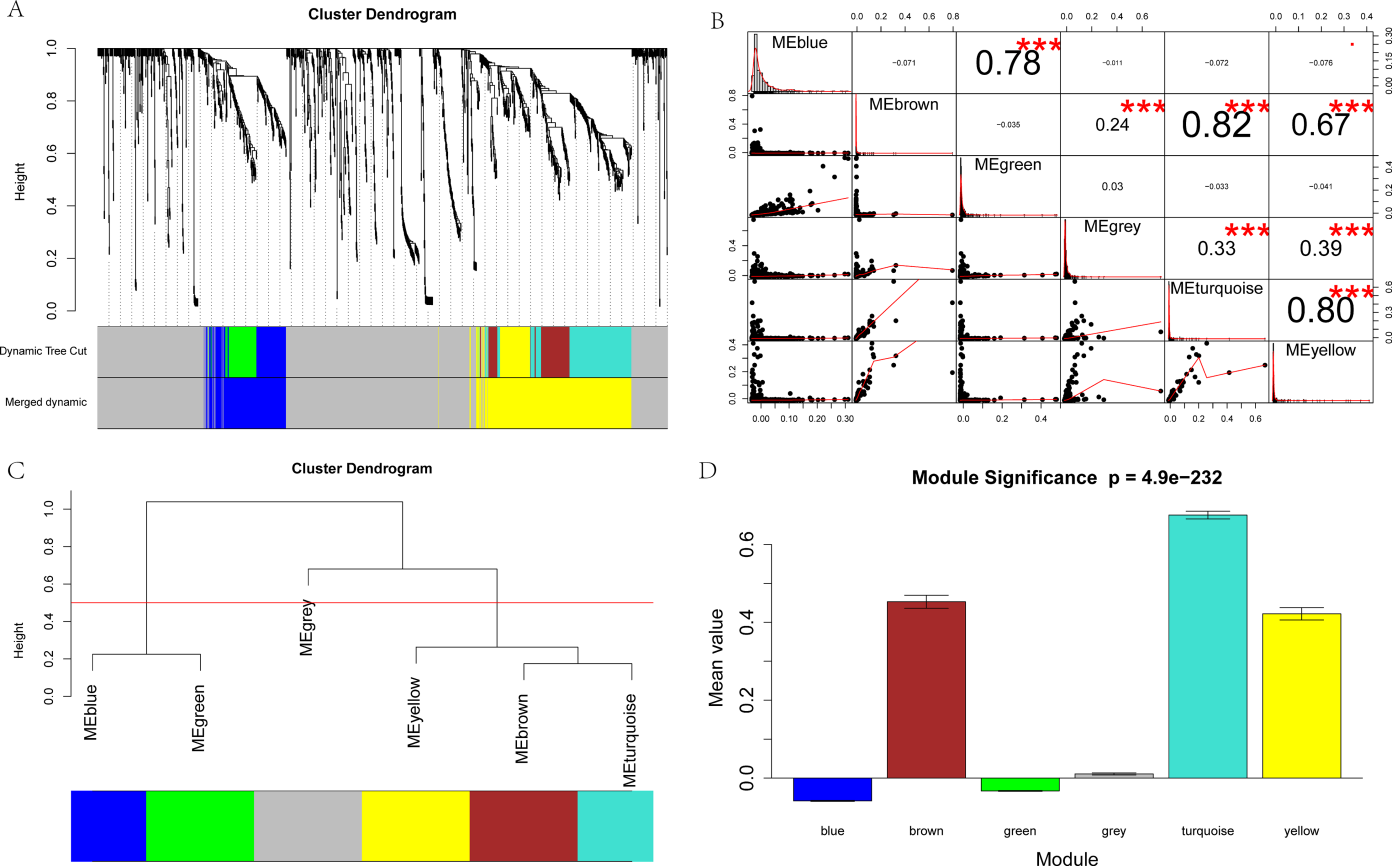
Co-expression analysis of DEGs. **A:** The network analysis of gene expression in malignant melanoma identified distinct modules of co-expression genes. Each leaf (short vertical line) in the dendrogram corresponds to a gene, and the branches are expression modules of highly interconnected groups of genes with a color to indicate the module assignment. **B:** Correlation between gene modules. * P < 0.05, ** P < 0.01, *** P < 0.001. **C:** Clustering of module eigengenes. These 6 modules yielded two main clusters. **D:** Module significance of the correlations between gene modules (P = 4.9e-232)).

The dimensionless topological network follows the power law and the soft threshold power (β), which is used to scale the adjacency matrix [19]. In this study, as shown in **Fig 2A**, six gene modules were partitioned by dynamic tree cutting. Each module is independently verified to the other modules based on the correlation and significance between each two modules (**Fig 2B**). After examining their eigengene co-expression, we merged these dynamic modules into three with a threshold of 0.5, which confirmed the reliability of the module divisions (**Fig 2C**). The module significance of the correlation between the modules is P = 4.9e-232.

As a key component of the detection module, the eigengene is the main indicator of the change in expression profile in the module, and its relevance to the phenotypes can help reveal key biological functions and identify potential biomarkers. By merging eigengenes, we found two main modules, shown in blue and yellow, worthy of further exploration.

### GO and KEGG enrichment analysis of blue and yellow module genes

By exploring the biological functions of the blue and yellow modules, we have carried out the analysis of gene ontology (GO) terms and the pathway analysis of the KEGG database. All the significant terms of the annotation systems are represented as color bars to compare the relative significance of the enriched terms, where the length and color saturation of each term are proportional to the gene count/ratio and the adjusted P-value obtained from the enrichment analysis.

**Fig 3:**
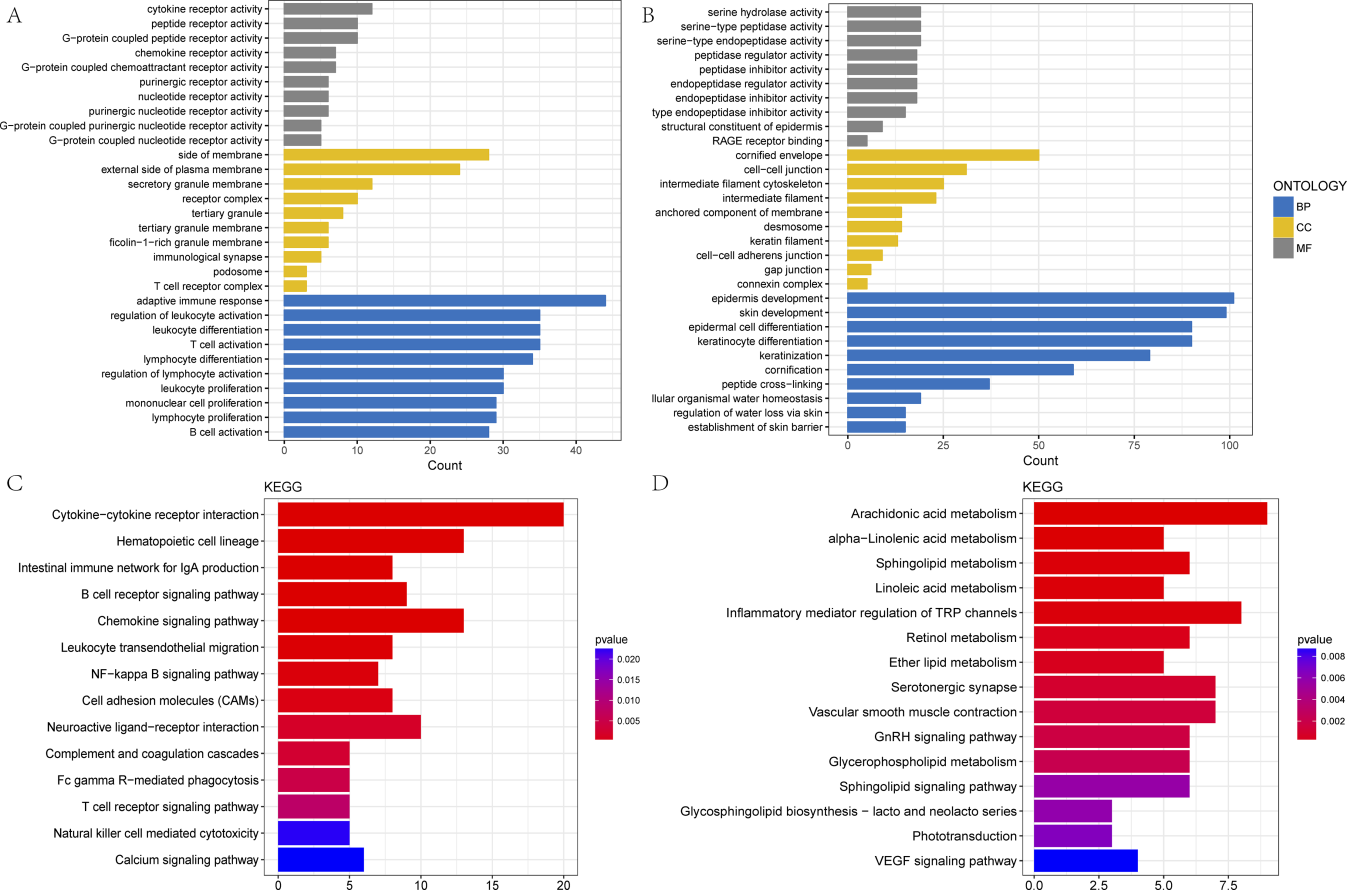
GO and KEGG enrichment of the blue and yellow modules. **A, B:** The top 30 enriched GO categories of biological process (BP), cellular component (CC) and molecular function (MF) in the blue and yellow modules; **C, D:** The top 15 enriched KEGG pathways in the blue and yellow modules. The magnitude of gene counts compared to all the background genes is represented by the horizontal bar length. The significance levels are represented by the legend’s color saturation.

In the blue module, the most GO terms in biological processes were associated with immune activities, including lymphocyte and leukocyte differentiation, proliferation and activation as well as T-cell and B-cell activation (**Fig 3A, Table 2**). The yellow module was enriched in keratinocyte and epidermis cell differentiation, keratinization and cornification, and the establishment of the skin barrier (**Fig 3B, Table 2**).

**Table 2:** Gene ontology (GO) enrichment analysis in blue and yellow modules (top 10 significantly enriched biological process, molecular function and cellular component).

ONT	Blue module			Yellow module		
	ID	Description	P_value	ID	Description	P_value
BP	0002250	adaptive immune response	1.15E-30	0030216	keratinocyte differentiation	1.83E-82
	0030098	lymphocyte differentiation	1.41E-25	0043588	skin development	4.61E-80
	0070661	leukocyte proliferation	5.09E-23	0008544	epidermis development	6.28E-78
	0046651	lymphocyte proliferation	1.47E-22	0031424	keratinization	1.75E-77
	0032943	mononuclear cell proliferation	1.84E-22	0009913	epidermal cell differentiation	1.29E-75
	0042110	T cell activation	1.72E-21	0070268	cornification	6.27E-71
	0042113	B cell activation	3.07E-21	0018149	peptide cross-linking	3.56E-48
	0002521	leukocyte differentiation	3.78E-21	0061436	establishment of skin barrier	5.34E-22
	0002694	regulation of leukocyte activation	2.09E-20	0033561	regulation of water loss via skin	5.66E-21
	0051249	regulation of lymphocyte activation	3.06E-17	0050891	multicellular organismal water homeostasis	1.52E-17
MF	0004896	cytokine receptor activity	1.09E-10	0030280	structural constituent of epidermis	1.08E-12
	0001637	G-protein coupled chemoattractant receptor activity	4.99E-09	0004867	serine-type endopeptidase inhibitor activity	9.10E-10
	0004950	chemokine receptor activity	4.99E-09	0004866	endopeptidase inhibitor activity	1.54E-08
	0001614	purinergic nucleotide receptor activity	4.41E-08	0061135	endopeptidase regulator activity	2.64E-08
	0016502	nucleotide receptor activity	4.41E-08	0030414	peptidase inhibitor activity	3.43E-08
	0035586	purinergic receptor activity	1.39E-07	0061134	peptidase regulator activity	5.82E-07
	0001608	G-protein coupled nucleotide receptor activity	1.76E-07	0004252	serine-type endopeptidase activity	1.07E-06
	0045028	G-protein coupled purinergic nucleotide receptor activity	1.76E-07	0050786	RAGE receptor binding	1.49E-06
	0008528	G-protein coupled peptide receptor activity	6.37E-07	0008236	serine-type peptidase activity	3.75E-06
	0001653	peptide receptor activity	6.84E-07	0017171	serine hydrolase activity	4.40E-06
CC	0009897	external side of plasma membrane	2.42E-16	0001533	cornified envelope	3.16E-73
	0098552	side of membrane	4.06E-14	0030057	desmosome	8.73E-18
	0001772	immunological synapse	2.08E-05	0045111	intermediate filament cytoskeleton	1.90E-11
	0101003	ficolin-1-rich granule membrane	3.86E-05	0005882	intermediate filament	2.13E-11
	0030667	secretory granule membrane	5.86E-05	0005911	cell-cell junction	5.67E-10
	0070821	tertiary granule membrane	0.000107	0045095	keratin filament	1.00E-07
	0070820	tertiary granule	0.000307	0031225	anchored component of membrane	7.53E-06
	0042101	T cell receptor complex	0.000928	0005921	gap junction	3.12E-05
	0043235	receptor complex	0.00189	0005922	connexin complex	4.66E-05
	0002102	podosome	0.004303	0005913	cell-cell adherens junction	7.06E-05

ONT: ontology; BP: biological process; MF: molecular function; CC: cellular component.

In the cellular component categories, the enriched GO terms in the blue module were primarily associated with receptor complexes, cell or granule membranes and immunological synapses (**Fig 3A, Table 2**). The yellow module genes were significantly related to connexin and keratin as well as gap and cell-cell junctions (**Fig 3B, Table 2**). In the molecular function categories, the GO terms enriched in the blue module genes included cytokines, chemokines, nucleotides, purine and G-protein coupled nucleotides or peptides or purinergic receptor activities, while the yellow module included endopeptidase, peptidase and serine-type inhibitors or regulator activities (**Fig 3A and 3B, Table 2**).

**Fig 4:**
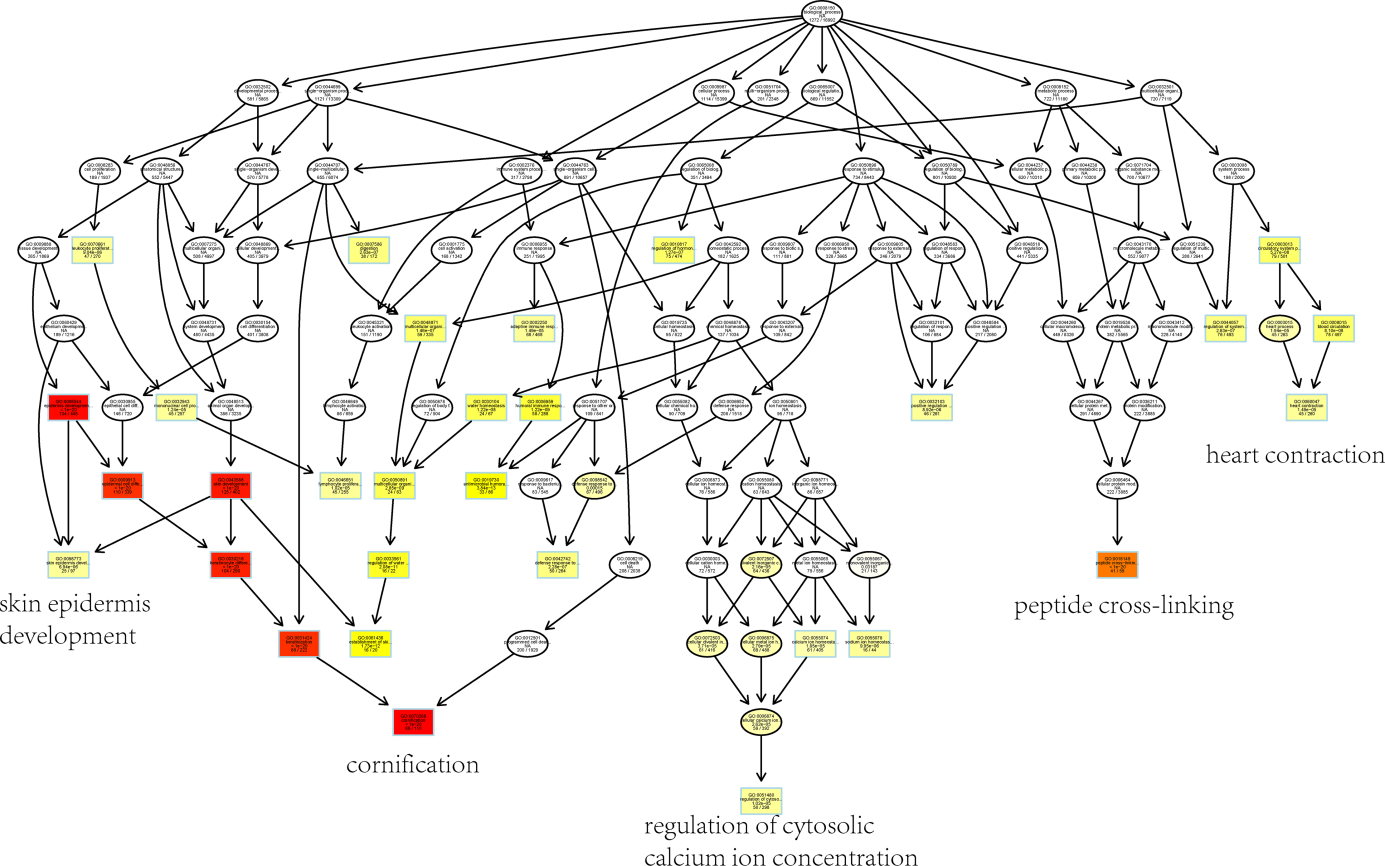
Cascade map of enriched biological processes. This cascade results in eight categories, including skin epidermis development, cornification, regulation of cytosolic calcium concentration, and peptide cross-linking. The color of the squares or circles represents a significant biological process with a criterion level P < 0.05; the significance levels are represented by the color saturation.

To explore the intrinsic cascade relationship between gene functions, we constructed a plot of gene functional regulation cascades for important GO terms in biological process (**Fig 4**). Then, we found that the genetic function between the hierarchical tree relationships was significant. The results showed that the gene functional cascade eventually induced keratinization and cornification (GO:0070268, *P*_adj_ = 1e-20), peptide cross-linking (GO:0018149, *P*_adj_ = 1e-20) and regulation of cytosolic calcium ion concentration (GO:0051480, *P*_adj_ = 1.02e-5).

To screen the significant pathway terms for the blue and yellow modules, the Fisher exact test was used as a statistical method for calculating the significance level (P-value < 0.05). According to the KEGG database, we obtained the pathways involved in all the genes of the blue and yellow modules by the annotating method. The blue module was mainly enriched with genes for cytokine-cytokine receptor interactions, intestinal immune network for IgA production, B-cell and T-cell receptor signaling pathways, NF-kappa B and chemokine signaling pathways (**Fig 3C, Table 3**). The yellow module was primarily enriched with genes involved in the metabolism of arachidonic acid, alpha-linolenic acid, linoleic acid, retinol and ether lipid as well as the GnRH, VEGF, IL-17 and Ras signaling pathways (**Fig 3D**, **Table 3)**.

**Table 3:** KEGG enrichment analysis of blue and yellow modules.

Blue			Yellow		
ID	Description	P_value	ID	Description	P_value
hsa04060	Cytokine-cytokine receptor interaction	3.32E-11	hsa00590	Arachidonic acid metabolism	1.37E-07
hsa04640	Hematopoietic cell lineage	9.26E-11	hsa00592	alpha-Linolenic acid metabolism	1.93E-05
hsa04672	Intestinal immune network for IgA production	9.91E-08	hsa00600	Sphingolipid metabolism	4.02E-05
hsa04662	B cell receptor signaling pathway	1.46E-07	hsa00591	Linoleic acid metabolism	4.13E-05
hsa04062	Chemokine signaling pathway	2.65E-07	hsa04750	Inflammatory mediator regulation of TRP channels	5.16E-05
hsa04670	Leukocyte transendothelial migration	5.65E-05	hsa00830	Retinol metabolism	0.000254
hsa04064	NF-kappa B signaling pathway	0.000139	hsa00565	Ether lipid metabolism	0.000357
hsa04514	Cell adhesion molecules (CAMs)	0.000343	hsa04726	Serotonergic synapse	0.000899
hsa04080	Neuroactive ligand-receptor interaction	0.00182	hsa04270	Vascular smooth muscle contraction	0.001345
hsa04610	Complement and coagulation cascades	0.002592	hsa04912	GnRH signaling pathway	0.001625
hsa04666	Fc gamma R-mediated phagocytosis	0.004779	hsa00564	Glycerophospholipid metabolism	0.002131
hsa04660	T cell receptor signaling pathway	0.008039	hsa04071	Sphingolipid signaling pathway	0.005643
			hsa04370	VEGF signaling pathway	0.008719
hsa04020	Calcium signaling pathway	0.022675	hsa04657	IL-17 signaling pathway	0.009043
			hsa04014	Ras signaling pathway	0.036992

### Identification of hub genes and PPI network construction

These two important modules, blue and yellow, contained 200 and 400 genes, respectively. The highly connected nodes in each module group were extracted as the hub genes. In each network, the node size and color depth are proportional to the connect strength (the sum of the degrees in the module).

**Fig 5:**
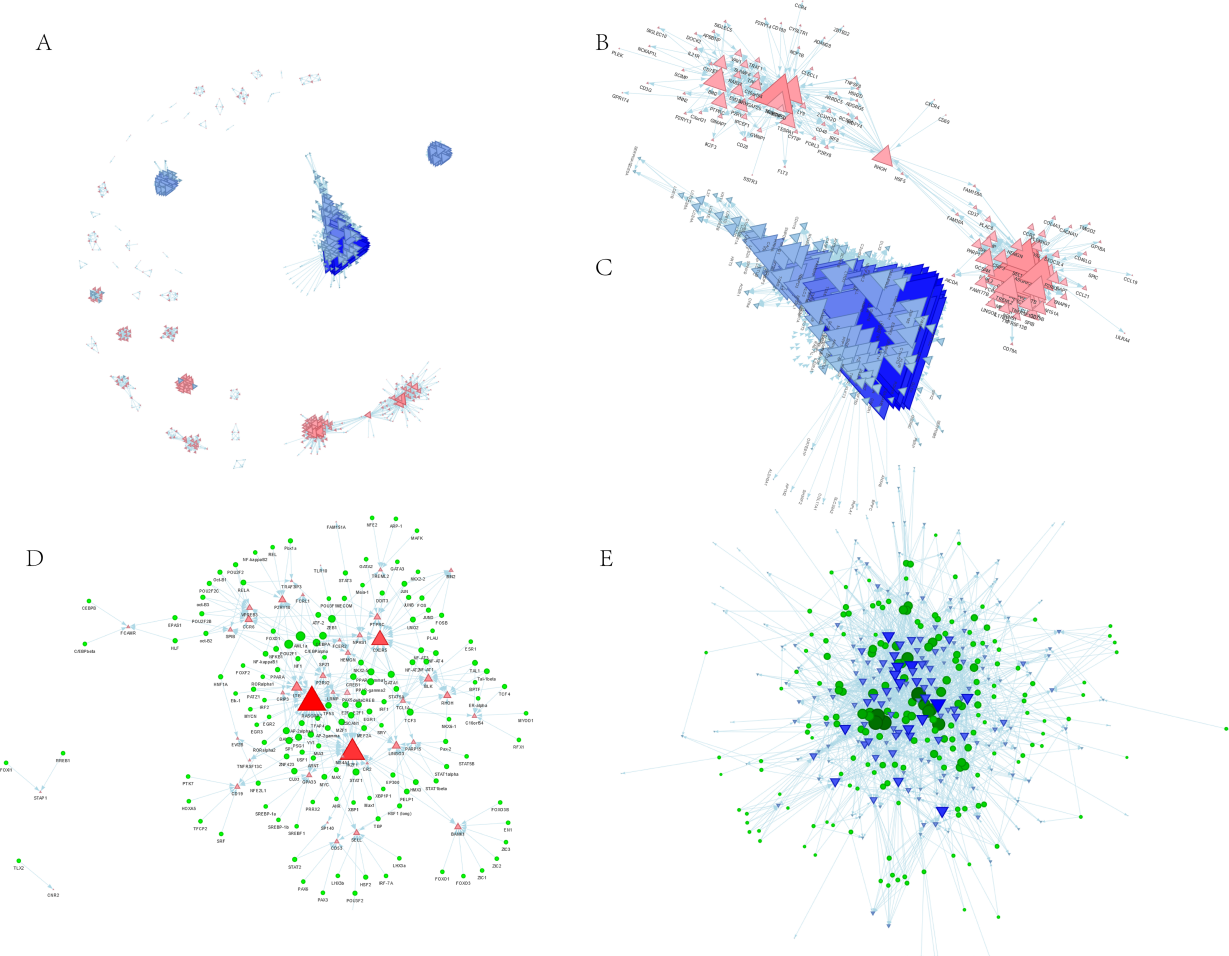
Transcription regulatory network of gene modules. **A:** PPI network of DEGs. **B:** hub genes in **the** subnetwork of the blue module. **C:** Hub genes in the subnetwork of the yellow module. **D:** The transcriptional regulators of the main hub genes of the blue module. **E:** The transcriptional regulators of the main hub genes of the yellow module. The triangle represents the DEGs in each enriched module, the red color and the up-direction represent up-regulated DEGs; the blue color and the down-direction represent down-regulated DEGs. The size of the nodes was weighted by the power of the DEGs that interacted with transcriptional regulators.

To compare and integrate our gene co-expression network-protein interaction data, we extracted a high-quality PPI network containing 705 nodes and 12,738 edges from the STRING database (**Fig 5A**). A node represents a gene in the network, and the edge represents the interaction between genes. Subsequently, we found the subnetworks in the STRING network genome of all the modules and extracted the central genes from the corresponding subnetworks. As shown in **Fig 5B**, the blue module subnetwork contains 123 nodes and 885 edges, whereas in **Fig 5C**, the yellow module subnetwork contains 274 nodes and 8,911 edges. Since the subnetworks were extracted from a high-quality STRING protein interaction database, the data indicate that these module genes have a tight regulatory relationship. Finally, 50 and 206 hub genes were found in the blue and the yellow module subnetworks, respectively.

### Transcriptional regulatory network

To identify genes that are most relevant to immunity and keratinization or cornification, we constructed a transcriptional regulatory network of hub genes in the two modules, blue and yellow, independently. Finally, nine hub-hub genes with degrees greater than 10 were extracted from the blue module: RASGRP2, IKZF1, CXCR5, LTB, BLK, LINGO3, CCR6, P2RY10 and RHOH (**Fig 5D, Table 4 and S1 Table**). In the yellow transcription network, fifteen hub-hub genes, including JUP, KRT14, PLA2G3, SPRR1A, KRT78, SFN, CLDN4, IL1RN, PKP3, CBLC, KRT16, TMEM79, KLK8, LYPD3 and LYPD5 were highly enriched in transcriptional regulators (**Fig 5E, Table 5 and S2 Table**). Additionally, several core transcriptional regulators were found to be mostly associated with our hub-hub genes, including GATA1, STAT1, SP1, PSG1, CUX1, TCF3, AML1a and POU2F1 in the blue module, and JUND, JUNB, JUN, FOSB, FOS, ZEB1, TCF3, TBP, MIA3, ARNT, AHR, FOSL1, EGR2, PAX5, GATA1, CAMSAP3, SP1, PSG1, DAND5, and CREB1, in the yellow module.

**Table 4:** Hub genes and connected transcriptional regulators in blue module.

Gene Symbol	Entrez Gene Name	logFC	P_value	Counts	TFs
RASGRP2	RAS guanyl releasing protein 2	2.088827	1.03E-06	39	AP-2alphaA, AP-2gamma, ARNT, CREB1, DAND5, E2F, E2F1, EGR1, EGR2, EGR3, IRF1, IRF2, MAX, MEF2A, MIA3, MYC, MYCN, MZF1, NF-kappaB1, NF1, NFKB1, NKX2-5, PATZ1, PAX5, POU2F1, PPAR-gamma1, PPAR-gamma2, PPARA, PSG1, RORalpha1, RORalpha2, SP1, SPZ1, USF1, YY1, ZEB1, ZNF423, ZSCAN1, deltaCREB,
IKZF1	IKAROS family zinc finger 1	1.627103	0.00026	29	AHR, AP-2alphaA, AP-2gamma, ARNT, CREB1, CUX1, DAND5, E2F, E2F1, EGR1, EP300, HMX3, MIA3, MYC, MZF1, Max1, PAX5, PELP1, PSG1, SP1, TFAP4, TP53, USF1, XBP1, XBP1P1, YY1, ZNF423, ZSCAN1, deltaCREB,
CXCR5	C-X-C motif chemokine receptor 5	2.765785	4.34E-08	23	AML1a, ATF-2, C/EBPalpha, CEBPA, CREB1, DDIT3, FOS, FOSB, GATA1, JUN, JUNB, JUND, LMO2, NF-AT1, NF-AT2, NF-AT3, NF-AT4, PLAU, PPAR-gamma1, PPAR-gamma2, STAT5A, TCF3, deltaCREB,
LTB	lymphotoxin beta	1.467307	0.00969	14	C/EBPalpha, CEBPA, DAND5, Elk-1, FOXD1, FOXF2, MZF1, PATZ1, PAX5, POU2F1, PSG1, SP1, TFAP4, ZSCAN1,
BLK	BLK proto-oncogene, Src family tyrosine kinase	2.791161	5.74E-08	11	BPTF, ER-alpha, ESR1, LMO2, MEF2A, PPAR-gamma1, PPAR-gamma2, STAT5A, TAL1, TCF3, Tal-1beta,
LINGO3	leucine rich repeat and Ig domain containing 3	1.615729	0.001714	11	EGR1, GATA1, HMX3, HSF1 (long), MZF1, PELP1, Pax-2, STAT1, STAT1alpha, STAT1beta, ZSCAN1,
CCR6	C-C motif chemokine receptor 6	1.675378	0.000302	10	AML1a, ATF-2, HNF1A, Oct-B1, POU2F1, POU2F2, POU2F2B, POU2F2C, oct-B2, oct-B3,
P2RY10	purinergic receptor P2Y10	1.978882	1.54E-05	10	AML1a, C/EBPalpha, CEBPA, NF-kappaB1, NF-kappaB2, NFKB1, POU2F1, Pbx1a, REL, RELA,
RHOH	ras homolog family member H	1.973277	5.15E-06	10	IRF1, NF-AT1, NF-AT2, NF-AT3, NF-AT4, Pax-2, TAL1, TCF3, TCF4, Tal-1beta,

log2FC: log2 fold change; TFs: transcriptional regulators.

**Table 5:** Hub genes and connected transcriptional regulators in yellow module.

Gene Symbol	Entrez Gene Name	logFC	P_value	Counts	TFs
JUP	junction plakoglobin	-2.57736	4.45E-21	30	AHR, BACH1, BRIP1, C/EBPalpha, CEBPA, Cart-1, DAND5, EGR3, GATA1, GATA2, GATA3, IL10, MRPL36, MYOD1, MZF1, Meis-1, NF-kappaB1, NF1, NFKB1, PATZ1, PSG1, PTK7, Pax-2, SP1, SRF, TCF3, TGIF1, TP53, ZNF423, ZSCAN1,
KRT14	keratin 14	-6.78423	3.75E-36	29	AHR, ARNT, C/EBPalpha, C/EBPbeta, CEBPA, CEBPB, E2F, E2F-3a, E2F1, E2F2, E2F4, E2F5, EN1, HMX3, HOXA3, IRF-7A, IRF2, LUN-1, MIA3, NFE2L1, NRSF form 1, NRSF form 2, PELP1, SOX5, SOX9, SRY, STAT5A, YY1, ZEB1,
PLA2G3	phospholipase A2 group III	-5.47685	1.43E-45	29	ARID5B, CBFB, CP1A, CP1C, GCGR, GR-alpha, GR-beta, HOXA5, KLF12, LUN-1, NF-Y, NFYA, NFYB, NFYC, NR3C1, SREBF1, SREBP-1a, SREBP-1b, STAT1, STAT1alpha, STAT1beta, STAT2, STAT3, STAT4, STAT5A, STAT5B, STAT6, TCF3, TFAP4,
SPRR1A	small proline rich protein 1A	-6.94951	5.89E-49	21	ATF-2, BACH1, BACH2, BRIP1, EP300, FOS, FOSB, FOSL1, JUN, JUNB, JUND, MAFK, MRPL36, MYC, MYOD1, MZF1, Max1, NFE2, RNASEH2A, TCF3, ZSCAN1,
KRT78	keratin 78	-5.23051	1.56E-32	20	AHR, AP-2gamma, ARNT, ATF-2, BPTF, FOXO4, GCGR, GR-alpha, HNF-4alpha2, MAX, MIA3, MYC, MYCN, NR3C1, SREBF1, SREBP-1a, SREBP-1b, TCF3, USF1, ZEB1,
SFN	stratifin	-5.49767	1.98E-30	20	AHR, ARNT, E2F, E2F1, FOS, FOSB, GATA1, HNF1A, HOXA3, IRF1, IRF2, JUN, JUNB, JUND, MIA3, PPAR-gamma1, PPAR-gamma2, XBP1, XBP1P1, ZEB1,
CLDN4	claudin 4	-2.72458	3.83E-12	18	ATF-2, CREB1, E2F, E2F1, FOS, FOSB, FOSL1, HMX3, JUN, JUNB, JUND, NF1, PELP1, PTK7, Pax-2, RNASEH2A, SRF, deltaCREB,
IL1RN	interleukin 1 receptor antagonist	-2.14742	1.39E-09	17	BACH1, BACH2, BRIP1, C/EBPalpha, CEBPA, IL10, MAFK, MRPL36, NFE2, PTK7, REL, RORalpha1, SRF, STAT1, STAT2, STAT5A, TGIF1,
PKP3	plakophilin 3	-4.45827	2.72E-30	17	BACH1, BRIP1, E2F, E2F1, HMX3, MAFK, MRPL36, NF-kappaB1, NFE2, NFKB1, NRSF form 1, NRSF form 2, PAX5, PELP1, PPARA, RELA, TFCP2,
CBLC	Cbl proto-oncogene C	-5.57704	1.63E-42	13	AP-2alphaA, AP-2gamma, KLF12, LUN-1, MYC, Max1, PAX5, STAT1, STAT1alpha, STAT1beta, TCF3, ZBTB6, ZNF423,
KRT16	keratin 16	-7.13816	2.15E-38	13	BACH1, BACH2, BRIP1, COUP-TF1, GATA1, HNF-4alpha2, LUN-1, MRPL36, NRSF form 1, NRSF form 2, RREB1, TBP, YY1,
TMEM79	transmembrane protein 79	-1.81015	2.86E-14	12	CREB1, CUX1, E2F, E2F1, GATA1, LMO2, NF1, PPARA, Pax-2, YY1, ZEB1, deltaCREB,
KLK8	kallikrein related peptidase 8	-6.12745	1.31E-42	10	IRF-7A, IRF1, IRF2, MYCN, MZF1, PPAR-gamma1, PPAR-gamma2, REL, ZIC1, ZSCAN1,
LYPD3	LY6/PLAUR domain containing 3	-5.18269	1.45E-55	10	AP-2alphaA, AP-2gamma, CREB1, FOXD3, MZF1, PRRX2, TP53, ZNF423, ZSCAN1, deltaCREB,
LYPD5	LY6/PLAUR domain containing 5	-2.58737	5.63E-16	10	LUN-1, Meis-1, NF-kappaB1, NFKB1, POU3F2, POU3F2 (N-Oct-5a), POU3F2 (N-Oct-5b), RELA, ZIC1, ZIC3,

log2FC: log2 fold change; TFs: transcriptional regulators.

## Discussion

Melanoma is one of the most common skin malignancies, and its poor prognosis is associated with a high metastatic capacity [20]. Currently, although several molecular biomarkers have been identified in the progress of melanoma, the mechanism of cell metastasis development has not been completely characterized [21]. The main purpose of this study was to utilize a global approach to construct a co-expression network of DEGs that predicted candidate gene clusters and hub genes involved in the pathogenesis of metastatic melanoma.

The WGCNA algorithms are designed to clarify the relationships among genes and maintain consistency among all the samples; therefore, they can be used to determine the phenotype responsible for complex biological mechanisms. The WGCNA unsupervised hierarchical clustering approach avoids potential bias and subjectivity decisions caused by a previous selection of candidate genes associated with malignant melanoma metastasis. In our study, from the 1,568 DEGs, six significant gene modules were merged into blue and yellow modules with a cut threshold of 0.5.

In the blue module, fifty hub genes with a mean logFC 2.32 were extracted from the 200 up-regulated genes (**S1 Table**). These genes are mainly involved in immune response activities, including B-cell, T-cell, lymphocyte, leukocyte and mononuclear cell responses. Active immunotherapy is a promising anti-cancer strategy designed to trigger specific T-cell responses against tumor cells to avoid relapse or progression of the disease. C-X-C chemokine receptor type 5 (CXCR5) is also one of the hub genes enriched in the cytokine-cytokine receptor pathway. CXCR5, known as CD185 or Burkitt lymphoma receptor 1 (BLR1), belongs to the CXC chemokine receptor family of the G protein coupled seven transmembrane receptors. It allows T cells to migrate to the lymph node B cell region [21]. Recently, the overexpression of CXCR5 in breast and prostate cancer patients was found to be highly correlated with lymph node metastasis and infiltration [22,23]. Elevated CXCR5 expression may lead to the survival and migration of abnormal cells in breast cancers deficient in functional p53 [24]. In addition, CXCR5 overexpression initiates a congenital and adaptive immune response through T-cells and B-cells [25].

For the 400 down-regulated genes in the yellow module, 206 hub genes with a mean logFC of -4.02 were identified as significant (**S2 Table**). These genes have a great impact on skin epidermal development and keratinization. The keratin families, including KRT 2, KRT 5, KRT 6, KRT 14, KRT 16, KRT 37, KRT 75, KRT 78 and KRT 80, have been shown to be significantly down-regulated during keratinocyte and epidermis development and in cytoskeleton intermediate filament termination. The main function of human melanocytes is to produce melanin for the epidermis to prevent damage from UVR [26,27]. The major structural proteins of epithelial cells, the keratins, are components of the intermediate filaments, and are the most heterogeneous of all the intermediate filaments [28,29]. The expression of KRT5 / KRT14 pairs is closely related to the intermediate phenotype of cells that undergo the epithelial-mesenchymal transition (EMT), which has a significant effect on tumor progression and metastasis because it helps to spread tumor cells throughout the body. Thus, paired KRT5 / KRT14 can be used to identify basal cell metastases [30].

Tyrosine is the precursor of the synthetic pigment melanin, which is a natural barrier to UVR damage but will weaken the effects of radiotherapy or chemotherapy. Therefore, melanin and melanogenesis are considered a double-edged sword [31]. Tyrosine and L-DOPA are recognized as substrates and intermediates of melanin biosynthesis. Studies have shown that increasing the supply of L-tyrosine in melanoma/melanocyte cell lines leads to an increase in melanoma cells [32]. In addition, L-tyrosine and L-DOPA are essential for the eventual formation of melanosomes in the pigment synthesis system of normal and malignant melanocytes as important mediators of cellular processes that affect not only the state of the melanocytes but also the recipient cells they affect [33]. Tyrosinase activity was tyrosine dose-dependent in melanotic and hypomelanotic cell lines, whereas tyrosinase activity continued to increase but slowed when melanization reached a plateau in amelanotic cells [34]. Recently, a study by Sarna et al. [35] showed that the presence of melanin affects the elasticity of cells as well as the movement of malignant melanocytes, which may help to understand the metastatic process of malignant melanomas. Our GO enrichment results revealed a few inhibited cellular component pathways, including gap junction (GO:0005921), fascia adherens (GO:0005916), connexin complex (GO:0005922), cell-cell junction (GO:0005911) and anchored component of membrane (GO:0031225), which may induce the metastatic ability of malignant melanocytes.

Interestingly, recent studies have shown that melanin levels in primary tumors increase with the development of metastasis and are high in deep melanoma cells [36]. Those studies also showed that the pigmentation increased with the local development of advanced melanoma and distant metastasis [37]. Additional evidence suggested that high melanin levels also contribute to hypoxia, dramatically changing the radiosensitivity of tumor cells and thus affecting the response of melanoma cells to antineoplastic agents [38]. Moreover, other evidence indicated that inhibitors of melanogenesis, including tyrosinase inhibitors, could reverse the immunosuppressive properties and chemoresistance of melanin [39]. However, they also observed the highest level of melanin in pT1-pT2 primary tumor melanomas [36]. Our results show that melanin biosynthesis was inhibited in the tyrosine metabolic pathway (ID: hsa00350) of metastatic melanoma. This inhibition could reflect the mechanism by which melanoma acquires a more malignant phenotype and resistance to therapy. The inhibition of melanogenesis may be an effective therapeutic strategy for melanotic melanomas.

In addition, during the initial formation of a tumor, immune suppression promotes tumor growth and progression. Our study found that genes involved in the biological processes of desmosome organization (GO: 0002934) and hemidesmosome assembly (GO: 00031581) in metastatic melanomas, including JUP, PKP3, KRT14, and KRT5, were inhibited. The reduction in the expression of these genes may play a critical role in altering tumor cell motility and, therefore, in increasing tumor cell metastasis. In addition, studies have shown that melanin granules formed in melanosomes, essential organelles that affect melanocyte elasticity, can inhibit melanoma cell migration [35]. Considering this evidence, we can infer that abnormalities in melanin metabolic activity, melanosomes and desmosomes are fundamental factors affecting the migration of melanoma cells, a finding that may contribute to a better understanding of the mechanism of malignant melanoma metastasis.

Tumor cell metastasis involves a series of complex factors that can migrate through the bloodstream or the lymphatic system, or directly to other parts of the body. Transcription factors play important roles in the cell movement that is the basis of metastasis formation. CREB (cAMP response element-binding protein) and its associated proteins (e.g., MMP-2, MCAM and MUC18) play a key role in tumor growth and metastasis of human melanomas [40]. Our results suggest that CREB is a transcriptional regulator of TGM1 (transglutaminase 1),LYPD3 (LY6/PLAUR domain containing 3), TMEM79 (transmembrane protein 79), and claudin 4. The activation transcription factors, which regulate SPRR1A (small proline rich protein 1A), KRT78, claudin 4 and RAB25 (a member of the RAS oncogene family), also play crucial roles in malignant melanoma metastasis [41]. However, their precise functions in metastatic melanoma have not been characterized.

## Conclusions

In summary, from 471 gene chip samples, through WGCNA analysis, we identified 1,486 DEGs consisting of two major gene co-expression modules. The results provide important insights into human malignant melanoma immunity and keratinocyte dysfunction. In addition, by constructing a transcriptional regulatory network, we have identified several major transcription factors, including GATA1, STAT1, SP1, and PSG1, that regulate the expression of 24 hub-hub genes. These genes may be the focus of subsequent work. These transcription factors may act as crucial regulators affecting gene expression by post-transcriptional or epigenetic modification. In addition, some of the hub-hub genes, which have not been reported before, showed important interactions in several pathways affecting biological processes, molecular mechanisms and cellular components. Our results potentially contribute to the better understanding of the progression of malignant melanoma as well as the identification of biomarkers for the early diagnosis of melanoma.

## Supporting information

S1 Table Hub genes and connected transcriptional regulators in blue module. (XLSX)

S2 Table Hub genes and connected transcriptional regulators in yellow module. (XLSX)
